# MSC-EVs alleviate osteoarthritic joint pain and degeneration by suppressing IL-1β/NGF signaling

**DOI:** 10.1016/j.gendis.2025.101981

**Published:** 2025-12-11

**Authors:** Kristeen Ye Wen Teo, Chengming Wen, Raymond Chung Wen Wong, Wei Seong Toh

**Affiliations:** a Department of Orthopaedic Surgery, Yong Loo Lin School of Medicine, National University of Singapore, Singapore 119228, Singapore; b Faculty of Dentistry, National University of Singapore, Singapore 119085, Singapore; c Tissue Engineering Program, Life Sciences Institute, National University of Singapore, Singapore 117510, Singapore

Temporomandibular joint osteoarthritis (TMJOA) is an important subtype of temporomandibular disorders characterized by progressive cartilage degradation, subchondral bone remodeling, synovial inflammation, chronic pain, and joint crepitus, adversely affecting the quality of life.^1^ Current management with non-steroidal anti-inflammatory drugs, intra-articular hyaluronic acid injections, or arthrocentesis reduces pain and inflammation to some extent, but does not repair the joint, underscoring the need for effective disease-modifying therapies.^1^

Mesenchymal stromal/stem cells (MSCs) and their derived extracellular vesicles (EVs) have demonstrated efficacy in the treatment of osteoarthritis (OA), including TMJOA, in clinical and animal studies. We previously reported that MSC-EVs were efficacious in alleviating TMJ joint pain and degeneration in a rat model.^2^ However, the molecular mechanisms remain to be fully clarified. In this study, we employed a rat model of TMJOA^2^ to investigate the mechanisms mediated by MSC-EVs in alleviating joint pain and degeneration. TMJOA was induced in rats by mono-iodoacetate (0.5 mg per 50 μL phosphate-buffered saline/PBS) injection (Fig. 1A). Two weeks after OA induction, three weekly intra-articular injections of MSC-EVs (1.3 × 10^10^ particles per 50 μL PBS) were given to OA+EVs rats, whereas equivalent PBS injections were given to OA+PBS rats. In sham animals, needle pricks were given. To gain insights into how MSC-EVs impact the protein phosphorylation and signaling networks in joint repair, PhosphoExplorer antibody array coupled with Ingenuity Pathway Analysis (IPA) was first performed to map the phosphorylated protein profile of the condylar cartilage (Fig. 1A). As depicted in Figure 1B, IPA analysis of the phosphorylated proteins showed distinct activation patterns across multiple signaling pathways with MSC-EV treatment. Notably, MSC-EVs activated several signaling pathways, including hypoxia-inducible factor 1-alpha (HIF-1α) and activin inhibin signaling pathways, which are involved in regulating chondrocyte survival, proliferation, differentiation, and matrix synthesis. These findings highlight the potential of MSC-EVs to enhance diverse chondrocyte functions that contribute to joint repair (Fig. 1B). On the other hand, the nerve growth factor (NGF) signaling pathway was among the pathways notably suppressed by MSC-EVs, as evidenced by a negative Z-score (Fig. 1B). This suppression implies that MSC-EVs may dampen NGF-mediated responses, commonly linked to pain sensitization and neurogenic inflammation, to alleviate OA pain and inflammation.

**Figure 1:**
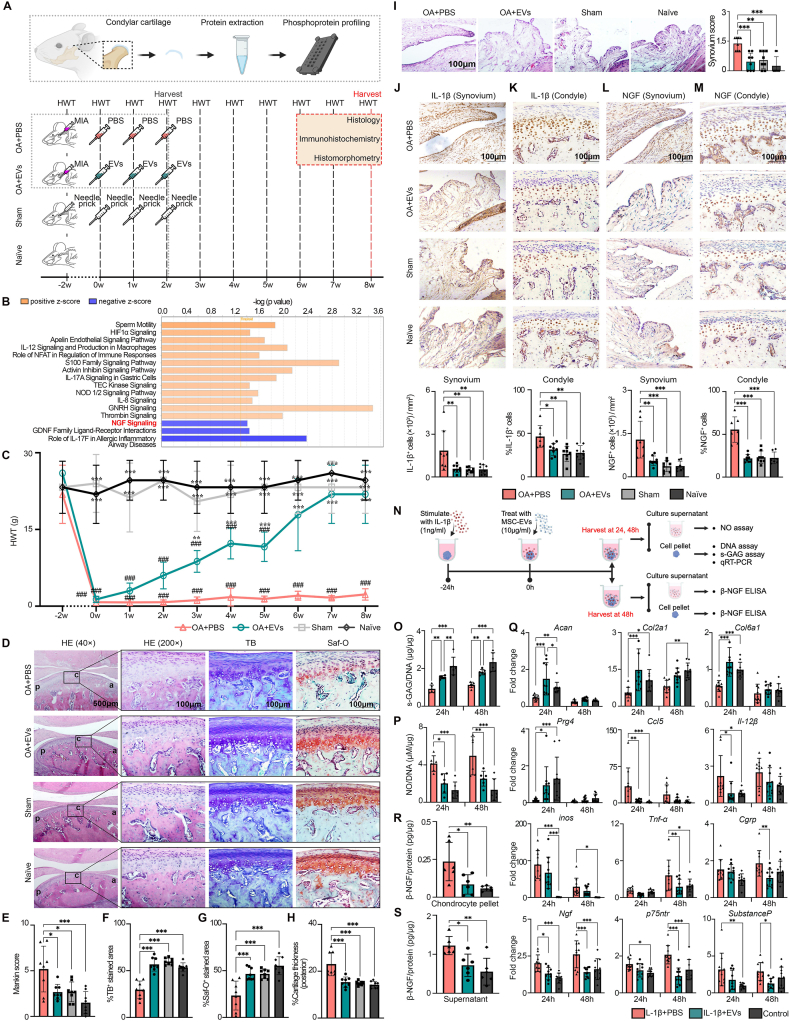
MSC-EVs alleviate osteoarthritis (OA) joint pain and degeneration by suppressing IL-1β/NGF signaling. **(A)** Schematic illustration of the *in vivo* animal study. To induce temporomandibular joint osteoarthritis, mono-iodoacetate (MIA, 0.5 mg per 50 μL PBS) was injected into the upper compartment of the temporomandibular joint. After 2 weeks of OA induction, rats were randomly divided to receive three weekly intra-articular injections of MSC-EVs (1.3 × 10^10^ particles per 50 μL PBS) for OA+EVs rats, or equivalent PBS injections for OA+PBS rats. Sham rats received needle pricks, whereas age-matched naïve rats served as unoperated controls. For phosphoproteomic analysis, temporomandibular joints from OA+EVs and OA+PBS rats were harvested 1 h after the third injection. Measurement of head withdrawal threshold (HWT) for nociceptive response was performed at weekly intervals. The rats were euthanized at 8 weeks post-treatment for analyses by histology, immunohistochemistry, and histomorphometry. **(B)** Ingenuity Pathway Analysis (IPA) predicted the top 16 canonical pathways from phosphorylated proteins of condyles treated with MSC-EVs as compared with PBS. Orange threshold line indicates –log (*P* value) at 1.3, corresponding to *P* < 0.05 using Fisher's exact test. **(C)** Nociceptive responses following treatment with MSC-EVs (*n* = 8 joints). Data were presented as mean ± standard deviation. Significance was determined using two-way ANOVA with a Bonferroni post hoc test. ^∗∗^*P* < 0.01 and ^∗∗∗^*P* < 0.001 compared with the OA+PBS group, and ^###^*P* < 0.001 compared with the Sham group. **(D)** Hematoxylin and eosin (HE), toluidine blue (TB), and Safranin-O (Saf-O) staining at 8 weeks. Representative images (*n* = 8 joints). Scale bars: 500 or 100 μm. **(E)** Mankin score. **(F, G)** Percentage areal deposition of s-GAG by quantification of (F) TB-stained area and (G) Saf-O-stained area. **(H)** Cartilage thickness was measured and expressed as a percentage of condylar height at the posterior region. Data were presented as mean ± standard deviation. Significance was determined using one-way ANOVA with a Bonferroni post hoc test. ^∗^*P* < 0.05 and ^∗∗∗^*P* < 0.001 compared with the OA+PBS group. **(I)** HE staining of the synovial membrane and synovial membrane inflammation score. Representative images (*n* = 8 joints). Scale bars: 100 μm. Data were presented as mean ± standard deviation. Significance was determined using one-way ANOVA with a Bonferroni post hoc test. ^∗∗^*P* < 0.01 and ^∗∗∗^*P* < 0.001 compared with the OA+PBS group. **(J**–**M)** Immunohistochemical staining and quantification of (J, K) IL-1β^+^ and (L, M) NGF^+^ cells in (J, L) synovium and (K, M) condylar cartilage. Representative images (*n* = 8 joints). Scale bars: 100 μm. Data were presented as mean ± standard deviation. Significance was determined using one-way ANOVA with a Bonferroni post hoc test. ^∗^*P* < 0.05, ^∗∗^*P* < 0.01, and ^∗∗∗^*P* < 0.001 compared with the OA+PBS group. **(N)** Schematic illustration of an *in vitro* chondrocyte OA model. Chondrocytes were stimulated with 1 ng/mL IL-1β for 24 h and then treated with MSC-EVs or PBS for 24 h and 48 h. **(O)** s-GAG/DNA quantification of chondrocyte pellets (*n* = 6). **(P)** Quantitative analysis of NO/DNA in chondrocyte culture supernatants (*n* = 6). **(Q)** Quantitative reverse transcription PCR analysis showed regulation of genes associated with cartilage matrix (*Acan*, *Col2a1*, *Col6a1*, and *Prg4*), inflammation (*Ccl5*, *Il-12β*, *Inos*, and *Tnf-α*), and pain (*Cgrp*, *Ngf*, *p75ntr*, and *Substance P*) by 10 μg/mL MSC-EV treatment (*n* = 10). Data were presented as mean ± standard deviation. Significance was determined using two-way ANOVA with a Bonferroni post hoc test. ^∗^*P* < 0.05, ^∗∗^*P* < 0.01, and ^∗∗∗^*P* < 0.001. **(R, S)** Measurement of β-NGF in (R) chondrocyte pellet and (S) culture supernatant (*n* = 6). Data were presented as mean ± standard deviation. Significance was determined using one-way ANOVA with a Bonferroni post hoc test. ^∗^*P* < 0.05, ^∗∗^*P* < 0.01, and ^∗∗∗^*P* < 0.001.

Besides being one of the key regulators of chronic OA pain, NGF and NGF receptors have also been implicated in OA pathogenesis.^3^^,^^4^ Jiang et al reported the presence of tropomyosin receptor kinase A (TrkA) and p75 neurotrophic receptor (p75NTR) as high- and low-affinity NGF receptors in human OA cartilage, and elevation of NGF expression in cultured chondrocytes stimulated by proinflammatory cytokine IL-1β.^3^ Another study by Yu et al found that the angiogenic activities of endothelial cells were up-regulated following co-culture with human chondrocytes in the presence of NGF, implicating the involvement of NGF signaling in angiogenesis of chondrocytes, possibly contributing to subchondral bone vascularization in OA.^4^ Therefore, NGF signaling represents an important pathway for therapeutic intervention in OA.

Next, we validated whether MSC-EVs suppressed NGF signaling to alleviate OA pain and degeneration in our *in vivo* animal model and *in vitro* chondrocyte cultures. Consistent with the reduced NGF signaling by MSC-EV treatment, pain behavioral analysis by head withdrawal threshold (HWT) measurements showed remarkable pain relief following MSC-EV treatment (Fig. 1C). Notably, OA+EV rats had reduced pain as evidenced by a higher HWT than that of OA+PBS rats as early as 3 weeks post-treatment (*P* = 0.002). The pain was gradually reduced to baseline, achieving the HWT close to that of sham (*P* > 0.999) and naïve rats (*P* > 0.999) by week 8 post-treatment (Fig. 1C). The pain relief was accompanied by improved joint repair and restoration (Fig. 1D–I). Relative to OA+PBS rats, OA+EV rats showed significant improvements in joint restoration, as evidenced by improved Mankin score (*P* = 0.018), increased sulfated glycosaminoglycan (s-GAG) deposition (*P* < 0.001), lesser cartilage thickening (*P* < 0.001), and reduced synovial inflammation (*P* < 0.001), and were comparable to that of sham and naïve animals (Fig. 1D–I).

Further immunohistochemical analysis showed that NGF was expressed in both synovium and condylar cartilage, similar to the expression of IL-1β, implicating the molecular interactions between NGF and IL-1β in mediating OA joint pain and degeneration (Fig. 1J–M). Consistent with the role of MSC-EVs in resolving inflammation and reducing pain, OA+EV rats had significantly lower numbers of IL-1β^+^ cells and NGF^+^ cells in both the synovium (*P* < 0.05) and condylar cartilage (*P* < 0.05) than the OA+PBS rats, and were comparable to those of sham and naïve animals (Fig. 1J–M).

To examine the molecular interactions between IL-1β and NGF, we applied an *in vitro* chondrocyte model of OA (Fig. 1N). In the presence of IL-1β, there was reduced s-GAG synthesis and elevated nitric oxide (NO) production, but these were reversed with MSC-EV treatment. Notably, MSC-EV treatment significantly increased the levels of s-GAG/DNA (*P* < 0.01; Fig. 1O) and decreased the levels of NO/DNA (*P* < 0.05; Fig. 1P) in IL-1β-stimulated chondrocytes at 24 h and 48 h. Consistent with the preserved s-GAG/DNA levels, we observed that MSC-EV treatment significantly up-regulated the expression of several genes associated with the cartilage matrix, including *Acan*, *Col2a1*, *Col6a1*, and *Prg4* as early as 24 h (*P* < 0.05; Fig. 1Q). In parallel with the suppressed NO production, we observed significant down-regulation in mRNA levels of proinflammatory mediators, namely *Ccl5* (*P* < 0.01) and *Il-12β* (*P* < 0.05) at 24 h and *Tnf-α* (*P* < 0.01) at 48 h in IL-1β-stimulated chondrocytes with MSC-EV treatment, as compared with the PBS treatment (Fig. 1Q). Additionally, we observed significant decrease in mRNA expression of neurotrophins and neuropeptides including *Ngf* at 24 and 48 h (*P* < 0.05) and *Cgrp* (*P* < 0.01), *p75ntr* (*P* < 0.001), and *Substance P* (*P* < 0.05) at 48 h under pro-inflammatory conditions induced by IL-1β in chondrocytes (Fig. 1Q). Concomitant with the reduced expression of pain-associated markers, we observed that MSC-EVs inhibited the IL-1β-induced NGF expression in chondrocytes. Notably, a significant reduction in NGF levels in IL-β-stimulated chondrocytes (∼50%; Fig. 1R) and their supernatants (∼40%; Fig. 1S) was observed, following MSC-EV treatment (*P* < 0.05), reaching a level close to the untreated control. These findings suggest that MSC-EVs can effectively alleviate the inflammatory and nociceptive response of chondrocytes under IL-1β-stimulated inflammation.

Beyond attenuating inflammation in chondrocytes, thereby reducing catabolic activity and NGF production, MSC-EVs may also influence other key cell types involved in OA pain and inflammation, including macrophages and sensory neurons.^5^ Specifically, MSC-EVs can promote M2-like macrophage polarization, which suppresses pro-inflammatory cytokines, such as IL-1β, which drives NGF expression. Moreover, MSC-EVs may directly inhibit NGF-induced hyperexcitability in sensory neurons, suggesting both indirect and direct mechanisms underlying their analgesic effects.^5^

Based on previous profiling of MSC-EV protein and miRNA cargoes, bioinformatics analysis revealed four proteins—IL10, ARHGDIA, APP, and RTN4—that may inhibit IL-1β/NGF signaling by interacting with the corresponding receptors, IL-1R1 and NGFR (Fig. S1A; Table S4). Additionally, five miRNAs (hsa-miR-765, hsa-miR-122-3p, hsa-miR-296-5p, hsa-miR-663a, and hsa-miR-1228-5p) were predicted to bind to the 3′ UTRs of both human *IL-1R1* or *NGFR*, and rat *Il-1r1* or *Ngfr* mRNAs, potentially suppressing the pathway activity (Fig. S1B and S1C; Table S5 and S6). However, these findings require further experimental validation.

Together, our findings demonstrate that MSC-EVs exert potent anti-inflammatory and analgesic effects in TMJOA by down-regulating the IL-1β/NGF signaling pathway—a central driver of nociception and joint degeneration.

## CRediT authorship contribution statement

**Kristeen Ye Wen Teo:** Writing – original draft, Visualization, Investigation, Formal analysis, Data curation. **Chengming Wen:** Formal analysis, Visualization, Writing – review & editing. **Raymond Chung Wen Wong:** Writing – review & editing. **Wei Seong Toh:** Writing – review & editing, Supervision, Funding acquisition, Conceptualization.

## Ethics declaration

All animal experiments were approved by the Institutional Animal Care and Use Committee at the National University of Singapore under protocol number: R18-1295.

## Funding

This work was supported by grants from the 10.13039/501100001349National Medical Research Council (Singapore) (No. CNIG23jul-0005, CNIG24jan-0001), the 10.13039/501100001352National University of Singapore (No. A-8001033-00-00), and the Ministry of Education (Singapore) (No. MOE-T2EP30122-0008).

## Conflict of interests

Wei Seong Toh is an Associate Editor for *Genes & Diseases* and was not involved in the editorial review or the decision to publish this article. Other authors declared no competing interests.
