# Supplementary material for: MSC-EVs alleviate osteoarthritic joint pain and degeneration by suppressing IL-1β/NGF signaling
Source: Genes Dis. 2025 Dec 11;13(3):101981. doi: 10.1016/j.gendis.2025.101981 (PMC12818981; doi:10.1016/j.gendis.2025.101981)
Supplement: Multimedia component 1 [file mmc1.docx]

**Materials and methods**

***Preparation and characterization of MSC-EVs***

MSC-EVs were prepared as described previously^1^. Briefly, immortalized E1-MYC 16.3 human embryonic stem cell-derived MSCs were cultured in Dulbecco’s Modified Eagle Medium (Thermo Fisher Scientific, Waltham, MA, USA) supplemented with 10% fetal bovine serum (Thermo Fisher Scientific). For EV preparation, the cells were cultured in a chemically defined culture medium for 3 days and the conditioned medium (CM) was harvested^2^. The CM was size fractionated and concentrated 50 × by tangential flow filtration using a membrane with molecular weight cut-off of 100 kDa (Sartorius, Göttingen, Germany). The EV preparation was characterized in accordance with the Minimal information for studies of extracellular vesicles (MISEV) 2018 guidelines^3^ and more specifically, with the identity and potency metrics proposed for MSC-derived small EV preparations^4;5^. Protein concentration was measured using the Pierce^TM^ Bradford protein assay (Thermo Fisher Scientific). Particle size distribution and concentration were analyzed using ZetaView (Particle Metrix). CD73/ecto-5´-nucleotidase activity was measured using PiColorLock Gold Phosphate Detection System (Innova Biosciences, Cambridge, UK). For this study, batch AC92 used was characterized to have a protein concentration of 1.011 μg/μl, particle concentration of 2.53 × 10^11^ particles/mg, particles of a modal size of 130.4 nm and CD73/ecto-5´-nucleotidase activity of 47.9 ± 0.512 mU/μg. The EV preparation was 0.22-μm filtered and stored in -20 °C freezer until further use.

***Animal experiments***

All procedures were performed according to Institutional Animal Care and Use Committee at the National University of Singapore under protocol number: R18-1295. A total of twenty Sprague-Dawley rats (8-week-old, female) with a mean weight of 225.5 ± 13.7 g (range 203-249 g) were used in this study. Briefly, monoiodoacetate (MIA, 0.5 mg/50 µl PBS was injected into the upper compartment of bilateral TMJs in 12 rats (24 TMJs). Two weeks after OA induction, OA+EVs rats (*n* = 12 TMJs) received three weekly intra-articular injections of MSC-EVs (1.3 × 10^10^ particles per injection), whereas OA+PBS rats (*n* = 12 TMJs) received equivalent PBS injections. This dosage of MSC-EVs was previously reported to be sufficient to promote TMJ repair^6^. Four sham rats (*n* = 8 TMJs) received needle pricks and another 4 non-injected animals (*n* = 8 TMJs) served as age-matched naïve controls. For phosphoprotein profiling, TMJs were harvested from rats in OA+PBS and OA+EV groups 1 h after the third EV injection. Four TMJs were collected from each group and pooled for analysis. All rats were housed under controlled temperature with a 12-h light/12-h dark cycle and allowed to move freely with access to food and water.

***Phosphoprotein profiling***

Total protein from the TMJ condylar cartilage was extracted using the Mammalian Tissue/Cell Extraction kit (BioVision, Milpitas, CA, USA). The extracted proteins were analyzed using the PhosphoExplorer antibody microarray, which contains a total of 1318 antibodies (Full Moon Biosystems, Sunnyvale, CA, USA) according to manufacturer’s instructions. Phosphorylation analysis of the proteins was performed using the Ingenuity Pathway Analysis (IPA) software (Qiagen, Hilden, Germany) to identify the biological processes, pathways and networks altered by MSC-EVs. Briefly, the phosphoprotein signals from MSC-EV-treated samples were compared to that of PBS-treated samples and calculated as ratio for each spot on the microarray slide. The ratios were then log-transformed (log_2_) and uploaded unto IPA for further analysis. “Core Analysis” was performed with threshold of signal => 1.4-fold change between OA+EV and OA+PBS condyles to predict the reasonable canonical pathways, upstream analysis and regulator effects. Pathways that met the criteria of activation z-score > |1.2| and -log(*P* value) > 1.3 were shown^7^. The directionality of the z-score was interpreted as follows: positive value indicates activation, while negative value indicates inhibition.

***Pain behavioral assessment***

Nociceptive responses of the rats were assessed using von Frey microfilament procedure as reported previously^8^. Briefly, the rats were tested with a series of filaments (Aesthesio®) starting from the lowest force at the preauricular area. The HWT is defined as the lowest force of the filaments that produced the withdrawal response for at least three times. Assessment of pain response was performed weekly and the HWT was calculated as a mean value per joint of 4 rats per group.

***Histology and immunohistochemistry***

Tissue samples were fixed in 10% (v/v) neutral buffered formalin for 1 week and then decalcified in 30% (v/v) of formic acid for another 2 weeks. Subsequently, the samples were dehydrated and embedded in paraffin for microtomy. Serial sections were cut at 5-µm thickness and stained with haematoxylin and eosin (HE) for general morphology, Safranin-O (Saf-O) and toluidine blue (TB) for sulphated glycosaminoglycan (s-GAG) deposition. Mankin scoring was performed by two independent blinded scorers to grade the joints using TB-stained sections according to parameters described in **Table S1**^9^, where score for normal cartilage is 0 and degenerated cartilage is 14. A synovial membrane inflammation scoring system^10^ was used by two independent blinded scorers to assess synovial inflammation based on number of synovial lining cell layers, proliferation of subsynovial tissue as well as infiltration of inflammatory cells (**Table S2**). The percentages of TB and Saf-O positively-stained areas for s-GAG were measured in the demarcated cartilage area at 40 × magnification using the ImageJ software (National Institutes of Health, Bethesda, MD, USA). To assess cartilage thickening, the cartilage thickness and condylar height at the posterior region on the condylar head were measured and calculated as the percentage of cartilage thickness against the condylar height. Immunohistochemistry was performed as previously described^6^ to detect interleukin (IL)-1β (Rabbit polyclonal, 1:1000, Abcam) and nerve growth factor (NGF) (Clone EP1320Y, 1:200, Abcam). Positively-stained cells in condylar cartilage were counted at 200 × magnification in three random fields and expressed as percentages of positive cells. Positively-stained cells in the synovium were counted at 200 × magnification and normalized by the tissue area (mm^2^).

**Table S1:** Mankin score

| **I. Structure** |  |
| --- | --- |
| a. Normal | 0 |
| b. Surface irregularities | 1 |
| c. Pannus and surface irregularities | 2 |
| d. Clefts to transitional zone | 3 |
| e. Clefts to radial zone | 4 |
| f. Clefts to calcified zone | 5 |
| g. Complete disorganization | 6 |
| **II. Cells** |  |
| a. Normal | 0 |
| b. Diffuse hypercellularity | 1 |
| c. Cloning | 2 |
| d. Hypocellularity | 3 |
| **III. Matrix staining** |  |
| a. Normal | 0 |
| b. Slight reduction | 1 |
| c. Moderate reduction | 2 |
| d. Severe reduction | 3 |
| e. No dye noted | 4 |
| **IV. Tidemark integrity** |  |
| a. Intact | 0 |
| b. Crossed by blood vessels | 1 |

**Table S2:** Synovial membrane inflammation score

| 1. No changes (1-2 layers of synovial lining cells) | 0 |
| --- | --- |
| 1. Increased number of lining cell layers (≥3-4 layers) or   slight proliferation of subsynovial tissue | 1 |
| 1. Increased number of lining cell layers (≥3-4 layers) and/or   proliferation of subsynovial tissue | 2 |
| 1. Increased number of lining cell layers (>4 layers) and/or   proliferation of subsynovial tissue and  infiltration of few inflammatory cells | 3 |
| 1. Increased number of lining cell layers (>4 layers) and/or   proliferation of subsynovial tissue,  infiltration of large number of inflammatory cells | 4 |

***In vitro chondrocyte model of OA***

*In vitro* chondrocyte model of OA was established as previously described^6^. Briefly, primary rat condylar chondrocytes were cultured at a density of 2 × 10^4^ cells/cm^2^ in DMEM/F12 (Hyclone) supplemented with 10% FBS, 1% PS and 25 μg/ml L-ascorbic acid-2-phophate (AA2P, Sigma, St. Louis, MO, USA). At passage (P) 2, cells were seeded in 96-well U-bottom plates at 2.5 × 10^5^ cells/well, and centrifuged at 300 g for 5 min to aggregate the cells. The resulting cell pellets were cultured in serum-free chondrogenic medium^11^ composed of high glucose DMEM (Hyclone) supplemented with 1% insulin-transferrin-selenium (ITS+1, Sigma), 1 mM sodium pyruvate (Thermo Fisher Scientific), 2 mM GlutaMAX™ (Thermo Fisher Scientific), 40 μg/ml L-proline (Sigma), 50 μg/ml AA2P, 10^−7^M dexamethasone (Sigma), 10 ng/ml transforming growth factor (TGF)-β1 (R&D Systems, Minneapolis, MN, USA) and 1% PS. The chondrocyte pellets were stimulated with 1 ng/ml IL-1β (PeproTech, Rocky Hill, NJ, USA) for 24 h, and then treated with 10 μg/ml of MSC-EVs or PBS for 24 and 48 h.

***Nitric Oxide, s-GAG and DNA measurements***

Nitric oxide (NO) in the pellet culture supernatants was measured using the Griess Reagent Kit (Thermo Fisher Scientific) according to manufacturer’s instructions. Briefly, 150 μl of standard or supernatant samples were incubated with 20 μl of Griess reagent and 130 μl of deionized water for 30 min at room temperature. The absorbance readings were taken at 548 nm using a microplate reader (Spark®, Tecan™, Männedorf, Switzerland). To measure s-GAG, cell pellets were digested with proteinase K digestion buffer (100 μg/ml in Tris-HCl buffer, pH 8) for 18 h at 60 °C and measured using Biocolor Blyscan Glycosaminolgycan Assay Kit (Biocolor Ltd, Carrickfergus, UK) according to the manufacturer’s instructions. DNA concentration of cell pellets was measured using Quant-iT™ Picogreen dsDNA assay kit (Thermo Fisher Scientific) with fluorescence readings taken at excitation 480 nm and emission 520 nm. The NO and s-GAG concentrations were normalized against total DNA content and presented as NO/DNA and s-GAG/DNA, respectively.

***Quantitative reverse transcription polymerase chain reaction (qRT-PCR)***

Total RNA was isolated from chondrocyte pellets using PureLink^TM^ RNA Mini kit (Thermo Fisher Scientific) following manufacturer’s protocol. The RNA was then reverse transcribed using iScript^TM^ Reverse Transcription Supermix (Bio-Rad Laboratories, Hercules, CA, USA) and amplified using CFX Connect^TM^ real-time PCR system (Bio-Rad) with iTaqTM Universal SYBR® Green Supermix (Bio-Rad), and primers as shown in **Table S3**. The PCR cycling condition comprised an initial denaturation at 95 °C for 30 s followed by 40 cycles of amplification consisting of 15 s at 95 °C and 30 s extension at 60 °C. Relative mRNA expression of targeted genes was normalized against glyceraldehyde 3-phosphate dehydrogenase (*Gapdh*) and calculated using comparative ∆CT method, and finally expressed as fold changes.

**Table S3:** Primer sequences

| Gene | Primer | Sequence (5´ to 3´) |
| --- | --- | --- |
| *Acan* | Forward | CAGAACCTTCGCTCCAATGAC |
|  | Reverse | CCTCAATGCCATGCATCACTT |
| *Ccl5* | Forward | CGTGAAGGAGTATTTTTACACCAGC |
|  | Reverse | CTTGAACCCACTTCTTCTCTGGG |
| *Col2a1* | Forward | CCCCTGCAGTACATGCGG |
|  | Reverse | CTCGACGTCATGCTGTCTCAAG |
| *Col6a1* | Forward | CCCTGGTGGACAAGGTGAAA |
|  | Reverse | CGCATGAGCCCTCTGATGAT |
| *Cgrp* | Forward | ATCTAAGCGGTGTGGGAATCTG |
|  | Reverse | TTCTTGCCAGGTGCTCCAAC |
| *Gapdh* | Forward | GGTCGGTGTGAACGGATTTGG |
|  | Reverse | GCCGTGGGTAGAGTCATACTGGAAC |
| *Il-12β* | Forward | CCGATGCCCCTGGAGAAAC |
|  | Reverse | CCTTCTTGTGGAGCAGCAG |
| *Inos* | Forward | GAAACTTCTCAGCCACCTTGG |
|  | Reverse | CCGTGGGGCTTGTAGTTGAC |
| *Ngf* | Forward | CACTCTGAGGTGCATAGCGT |
|  | Reverse | GGATGAGCGCTTGCTCCTGT |
| *p75ntr* | Forward | AGGGATGGCGTGACTTTC |
|  | Reverse | GTTGGCTTCAGGCTTATGC |
| *Prg4* | Forward | GAATGGTGAGTTCAGGCTCCTTAG |
|  | Reverse | CCCAAGACTACAGCGGCAAAAC |
| *Substance P* | Forward | TGGCGGTCTTTTTTCTCGTT |
|  | Reverse | GCATTGCCTCCTTGATTTGG |
| *Tnf-α* | Forward | CCAGGTTCTCTTCAAGGGACAA |
|  | Reverse | GGTATGAAATGGCAAATCGGCT |

***β-NGF ELISA and protein quantification***

β-NGF was measured in chondrocyte pellets and culture supernatants at 48 h following EV treatment using the rat β-NGF ELISA kit (Sigma) following the manufacturer’s instructions. The absorbance readings were obtained at 450 nm using the microplate reader (Spark®, Tecan^TM^). Total protein concentration of chondrocyte pellets and culture supernatants was measured using the Pierce^TM^ Coomassie (Bradford) protein assay kit (Thermo Fisher Scientific). The absorbance readings were taken at 595 nm using the microplate reader. The β-NGF concentrations were normalized against the total protein concentrations, and presented as β-NGF/Protein.

***Bioinformatics analysis of MSC-EV cargoes regulating IL-1β/NGF signaling***

MSC-EV protein and miRNA cargoes have been previously profiled^12;13^. For protein-protein interaction (PPI) analysis, the MSC-EV proteins and core receptors of IL-1β and NGF (IL-1R1 and IL-1RAP for IL-1β signaling, NGFR and NTRK1 for NGF signaling) were input into the STRING database (v12.0) for potential PPI searches. Obtained PPI items were then filtered using an interaction score higher than 0.9 as the filtering threshold. For miRNA target gene prediction, the obtained MSC-EV miRNAs were subjected to a batch analysis using TargetScan (Human v7.2), with a -0.2 context++ score threshold used to filter potential targets, where lower scores indicate higher predicted binding efficacy. For those miRNAs that may target any of the genes encoding IL-1β and NGF core receptors, their mature sequences were obtained from miRBase, and the miRNA-3'UTR binding potential of target genes (both human and rat sources) was cross-validated using the miRanda (v3.3a) database, with a filtering threshold of a score higher than 140 and a binding energy lower than -1.

***Statistical analysis***

Statistical analysis was performed using GraphPad Prism 10.2.0 (GraphPad Inc, San Diego, CA, USA). The data were reported as mean ± standard deviation (SD) and tested for normality. Statistical differences between the treatment groups were determined by one-way or two-way ANOVA followed by Bonferroni post hoc test for normally distributed data, and Kruskal-Wallis test followed by Dunn’s Multiple Correction Test for non-normally distributed data. In IPA analysis, Fisher’s Exact Test was used to assess the statistical significance of the association between the experimental dataset and the identified canonical pathways. The statistical significance was set as *P* < 0.05.

**Results**



**Figure S1** Identification of potential MSC-EV cargos in inhibiting the IL-1β/NGF signaling pathway. (A) PPI analysis targeting IL-1R1 and NGFR based on STRING database (v12.0). (B) miRNA binding prediction targeting the 3' UTRs of human *IL-1R1* and *NGFR,* and (C) rat *Il-1r1* and *Ngfr,* based on miRanda database (v3.3a).

| **Table S4:** PPI analysis based on STRING database (v12.0) | | | | | |
| --- | --- | --- | --- | --- | --- |
| **MSC-EV protein** | | **Target protein** | | **PPI Score** | **Effects**  **[Reference]** |
| **Protein Name** | **Location** | **Protein Name** | **Signaling Pathway** |  |  |
| IL-10 | Internal | IL-1R1 | IL-1β signaling | 0.917 | Inhibition^14^ |
| ARHGDIA | Internal | NGFR | NGF signaling | 0.992 | Inhibition^15^ |
| APP | Surface | NGFR | NGF signaling | 0.959 | Inhibition^16^ |
| RTN4 | Surface | NGFR | NGF signaling | 0.977 | Inhibition^17^ |

| **Table S5:** miRNA target gene prediction based on TargetScan database (human7.2) | | | | | |
| --- | --- | --- | --- | --- | --- |
| **miRNA** | **Target gene** | **3P-seq tags + 5** | **Total sites** | **Total context++ score** | **Note** |
| hsa-miR-765 | *IL-1R1* | 1713 | 3 | -0.34 |  |
| hsa-miR-122-3p | *IL-1R1* | 1713 | 1 | -0.25 |  |
| hsa-miR-663a | *NGFR* | 5 | 1 | -0.24 |  |
| hsa-miR-1228-5p | *NGFR* | 5 | 1 | -0.21 |  |
| hsa-miR-296-5p | *NGFR* | 5 | 4 | -0.68 | Conserved sites |

| **Table S6:** miRNA target gene prediction based on miRanda database (v3.3a) | | | | | |
| --- | --- | --- | --- | --- | --- |
| **miRNA** | **Gene** | **Species** | **Total Score** | **Total Energy** | **Total sites** |
| hsa-miR-765 | *IL-1R1* | Human | 155 | -22.61 | 1 |
| hsa-miR-122-3p | *IL-1R1* | Human | 145 | -12.83 | 1 |
| hsa-miR-765 | *NGFR* | Human | 292 | -37.86 | 2 |
| hsa-miR-296-5p | *NGFR* | Human | 303 | -47.43 | 2 |
| hsa-miR-663a | *NGFR* | Human | 295 | -56.6 | 2 |
| hsa-miR-1228-5p | *NGFR* | Human | 283 | -63.79 | 2 |
| hsa-miR-765 | *Il-1r1* | Rat | 420 | -50.42 | 3 |
| hsa-miR-1228-5p | *Il-1r1* | Rat | 299 | -51.28 | 2 |
| hsa-miR-1228-5p | *Ngfr* | Rat | 149 | -32.22 | 1 |
| hsa-miR-765 | *Ngfr* | Rat | 431 | -56.94 | 3 |
| hsa-miR-122-3p | *Ngfr* | Rat | 146 | -14.61 | 1 |
| hsa-miR-296-5p | *Ngfr* | Rat | 147 | -22.36 | 1 |
| hsa-miR-663a | *Ngfr* | Rat | 151 | -28.43 | 1 |

**References**

1. Chen TS, Arslan F, Yin Y, et al. Enabling a robust scalable manufacturing process for therapeutic exosomes through oncogenic immortalization of human ESC-derived MSCs. *J Transl Med.* 2011;9:47.

2. Sze SK, de Kleijn DP, Lai RC, et al. Elucidating the secretion proteome of human embryonic stem cell-derived mesenchymal stem cells. *Mol Cell Proteomics.* 2007;6(10):1680-1689.

3. Théry C, Witwer KW, Aikawa E, et al. Minimal information for studies of extracellular vesicles 2018 (MISEV2018): a position statement of the International Society for Extracellular Vesicles and update of the MISEV2014 guidelines. *J Extracell Vesicles.* 2018;7(1):1535750.

4. Witwer KW, Van Balkom BWM, Bruno S, et al. Defining mesenchymal stromal cell (MSC)-derived small extracellular vesicles for therapeutic applications. *J Extracell Vesicles.* 2019;8(1):1609206.

5. Gimona M, Brizzi MF, Choo ABH, et al. Critical considerations for the development of potency tests for therapeutic applications of mesenchymal stromal cell-derived small extracellular vesicles. *Cytotherapy.* 2021;23(5):373-380.

6. Zhang S, Teo KYW, Chuah SJ, Lai RC, Lim SK, Toh WS. MSC exosomes alleviate temporomandibular joint osteoarthritis by attenuating inflammation and restoring matrix homeostasis. *Biomaterials.* 2019;200:35-47.

7. Ouyang L, Chen Y, Wang Y, et al. p39-associated Cdk5 activity regulates dendritic morphogenesis. *Scientific Reports.* 2020;10(1):18746.

8. Ren K. An improved method for assessing mechanical allodynia in the rat. *Physiol Behav.* 1999;67(5):711-716.

9. Mankin HJ, Dorfman H, Lippiello L, Zarins A. Biochemical and metabolic abnormalities in articular cartilage from osteo-arthritic human hips. II. Correlation of morphology with biochemical and metabolic data. *J Bone Joint Surg Am.* 1971;53(3):523-537.

10. Gerwin N, Bendele AM, Glasson S, Carlson CS. The OARSI histopathology initiative - recommendations for histological assessments of osteoarthritis in the rat. *Osteoarthritis Cartilage.* 2010;18 Suppl 3:S24-34.

11. Toh WS, Liu H, Heng BC, Rufaihah AJ, Ye CP, Cao T. Combined effects of TGFbeta1 and BMP2 in serum-free chondrogenic differentiation of mesenchymal stem cells induced hyaline-like cartilage formation. *Growth Factors.* 2005;23(4):313-321.

12. Chen TS, Lai RC, Lee MM, Choo ABH, Lee CN, Lim SK. Mesenchymal stem cell secretes microparticles enriched in pre-microRNAs. *Nucleic Acids Research.* 2009;38(1):215-224.

13. Lai RC, Tan SS, Teh BJ, et al. Proteolytic Potential of the MSC Exosome Proteome: Implications for an Exosome-Mediated Delivery of Therapeutic Proteasome. *International Journal of Proteomics.* 2012;2012(1):971907.

14. Sun Y, Ma J, Li D, et al. Interleukin-10 inhibits interleukin-1β production and inflammasome activation of microglia in epileptic seizures. *J Neuroinflammation.* 2019;16(1):66.

15. Ramanujan A, Li Z, Ma Y, Lin Z, Ibáñez CF. RhoGDI phosphorylation by PKC promotes its interaction with death receptor p75(NTR) to gate axon growth and neuron survival. *EMBO Rep.* 2024;25(3):1490-1512.

16. Xu W, Weissmiller AM, White JA, 2nd, et al. Amyloid precursor protein-mediated endocytic pathway disruption induces axonal dysfunction and neurodegeneration. *J Clin Invest.* 2016;126(5):1815-1833.

17. Farrer RG, Kartje GL. Overexpression of Nogo-A changes nerve growth factor signaling dynamics in PC12 cells. *Cell Signal.* 2025;127:111569.
